# Challenges in Pediatric Foregut Replacements: An 11-Year Single-Center Experience

**DOI:** 10.7759/cureus.112162

**Published:** 2026-07-06

**Authors:** Susana Fortich, Jana E DeJesus, Esther Ewing, Geetha Radhakrishnan, Ravi Radhakrishnan

**Affiliations:** 1 Department of Surgery, University of Texas Medical Branch, Galveston, USA; 2 Department of Pediatrics, University of Texas Medical Branch, Galveston, USA; 3 Department of Pediatric Surgery, University of Texas Medical Branch, Galveston, USA

**Keywords:** colonic interposition, esophagectomy in children, foregut reconstruction, gastric interposition, pediatric esophageal replacement

## Abstract

Introduction

Foregut replacements are performed in the pediatric population for esophageal atresia, long-segment strictures resistant to repeated dilations, caustic injuries, and malignancy. The need for foregut replacements has declined due to advances in the management of esophageal atresia and GERD (gastroesophageal reflux disease), as well as protective devices on caustic chemical containers. However, given the technical challenges of these procedures, pediatric and general surgeons should be prepared in case a foregut replacement is required. This case series describes 10 foregut replacements performed between 2013 and 2024.

Methods

Case logs of a pediatric surgeon at an academic medical center were reviewed between December 2013 and December 2024. Cases involving “esophagectomy”, “esophageal replacement”, “ileocolonic interposition”, and “gastric pull-up” were included. Patient charts and operative reports were reviewed.

Results

A total of 10 pediatric patients (aged eight months to 23 years) underwent foregut replacement. Most patients were female (n = 6, 60%) and Hispanic (n = 8, 80%). Esophageal atresia and caustic injury were the leading indications for surgery. Ileocolonic conduits were the most commonly used reconstruction method (n = 6, 60%). Early complications included anastomotic leaks (n = 5, 50%), infections (n = 3, 30%), and dumping syndrome (n = 1, 10%). Late complications included dysphagia (n = 7, 70%), stricture formation (n = 5, 50%), and GERD (n = 4, 40%).

Conclusion

Though infrequently performed, foregut replacements in the pediatric population are still required in certain pathologic cases. Our series examined 10 patients who underwent replacement over 11 years. Ileocolonic conduits were the most frequently performed reconstruction, and late complications were more common. Understanding the patient’s clinical history and knowledge of conduit and route options are important for proper management.

## Introduction

The history of esophageal surgery is defined by landmark advancements that have shaped modern surgical management. In the 1870s, Theodor Billroth and Vincenz Czerny performed the first successful cervical esophageal resections without reconstruction [1]. This was followed by Jan Mikulicz-Radecki’s pioneering cervical esophagus reconstruction in 1886. In 1915, Franz J. A. Torek achieved the first transthoracic esophageal resection, opting not to reconstruct the esophagus but instead connecting the stomas externally with a 3-foot-long rubber tube, enabling the patient to eat for 17 more years [2].

Congenital esophageal atresia (EA) was first described by William Durston in 1670, with Thomas Gibson later documenting its association with tracheoesophageal fistula (TEF) in 1697 [3]. The first recorded surgical attempt to repair pure EA occurred in 1888 when Charles Steele performed a gastrotomy and attempted to pass a steel probe through the esophageal membrane. Significant progress came in 1939 when Logan Leven and William Ladd independently connected an esophagostomy to a gastrostomy to manage EA. A breakthrough followed in 1941 when Cameron Haight successfully performed a primary anastomosis by ligating the TEF and directly anastomosing the esophagus. Throughout the 1940s, William Ladd, Robert Gross, and Orvar Swenson further refined surgical techniques, developing esophageal replacement methods that dramatically improved survival rates for patients with EA, with or without TEF [4]. Ladd and colleagues emphasized that EA was no longer an incurable condition, underscoring the importance of establishing esophageal continuity for future interventions [5]. Their foundational work set the stage for modern esophageal reconstruction and ongoing advancements in surgical management. Today, assessing the need for foregut replacement requires a comprehensive evaluation of the patient’s anatomy, underlying pathology, and prior surgical history. Imaging studies, including contrast studies, endoscopy, and CT, are essential for surgical planning. In some cases, temporary measures such as cervical esophagostomy or mediastinal drainage may be necessary before definitive reconstruction.

In pediatric patients, esophagectomy and foregut replacement surgery are primarily indicated for long-gap EA, long-segment strictures refractory to dilatation, caustic injuries, and, in rare cases, malignancies. The global prevalence of EA ranges from 1 in 2,500 to 1 in 4,500 births, with an estimated incidence of 2.3 cases per 10,000 live births in the United States [6]. Although the incidence of caustic ingestion in children in the U.S. has been declining by 5,000 to 15,000 cases per year, it remains a significant concern, with an occurrence rate of 15.8 cases per 100,000 individuals [7]. Although malignant esophageal tumors are exceedingly rare in the pediatric population, they remain a potential indication for foregut replacement [8].

Advances in the management of EA and improvements in caustic injury prevention have reduced the frequency at which foregut replacements are performed. However, the continued incidence of refractory cases highlights the need for pediatric surgeons to remain prepared to perform these complex procedures. This case series examines 10 cases of esophageal replacement performed by the same pediatric surgeon at our institution over an 11-year period. We report the indications for operation, conduit selected, and outcomes, including early and late complications following surgery. A literature review was then performed to compare results with other pre-existing case series and expand the existing body of knowledge.

This article was previously presented as a meeting abstract at the 2025 SSAT Annual Scientific Meeting in May 2025.

## Materials and methods

This retrospective descriptive case series was conducted at the Department of Pediatric Surgery, The University of Texas Medical Branch, from July 2013 to June 2024. The charts of patients treated with esophageal replacement under the pediatric surgery service during the time period were reviewed through the electronic medical record (EMR) by three separate reviewers - one faculty pediatric surgeon and two general surgery residents. Cases were found by filtering EMR case logs with CPT codes 43107 (total or near-total esophagectomy with cervical esophagostomy, without thoracotomy), 43108 (total or near-total esophagectomy, without thoracotomy; with colon interposition or small intestine reconstruction), 43112 (total or near-total esophagectomy, with thoracotomy; with pharyngogastrostomy or cervical esophagogastrostomy, with or without pyloroplasty), or 43113 (total or near-total esophagectomy, with thoracotomy; with colon interposition or small intestine reconstruction, including intestine mobilization, preparation, and anastomosis). All patients were under 18 years of age, with the exception of one. This patient had achalasia and previously underwent a Heller myotomy and fundoplication at the age of 15 years but subsequently developed intractable dysphagia. She underwent esophagectomy and reconstruction at the age of 23 years with the pediatric surgery service.

Data extracted from chart review included demographics (e.g., age, gender, race, and ethnicity); presence of congenital anomalies; indications for esophageal replacement based on diagnosis via history and physical or preoperative progress notes; reconstruction type performed, delineated by operative note (e.g., gastric interposition, colonic interposition, ileocolonic interposition, or jejunal interposition); complications based on postoperative progress and follow-up notes, imaging studies, and laboratory data (immediate complications included anastomotic leak, bleeding, and infection; long-term complications included stricture, GERD (gastroesophageal reflux disease), dysphagia, and weight loss, which are the most commonly cited after foregut replacement); and functional outcomes based on postoperative follow-up notes and the number of endoscopic dilations needed for patients with stricture. Strictures were defined as symptomatic with dysphagia or PO intolerance, with radiographic evidence from an upper gastrointestinal series or computed tomography with oral contrast. Only anastomotic leaks requiring either percutaneous drainage or re-exploration were included as early infections. Follow-up data were collected up to five years out from the index operation. All data were collected into IBM SPSS Statistics for Windows, Version 29 (Released 2023; IBM Corp., Armonk, New York) in a de-identified manner. Descriptive statistics were generated (frequencies and percentages). This study was conducted in accordance with the institutional guidelines for human research. Approval was obtained from the Institutional Review Board of The University of Texas Medical Branch (IRB no. 25-0317). The authors declare no conflicts of interest and report that this research received no specific grant or financial support from any public, commercial, or not-for-profit funding agency.

Surgical technique

The four most commonly used methods of esophageal replacement still in practice include gastric interposition, ileocolic interposition, jejunal interposition, and colonic interposition. All types of esophageal replacements share common steps: selection and preparation of a graft, resection of a rudimentary or strictured esophagus, and establishment of continuity between the esophagus and the stomach.

Gastric Interposition

The stomach is the most commonly used conduit for esophageal replacement due to its robust submucosal vascular plexus, which enables extensive mobilization and a reliable blood supply. The standard gastric pull-up (gastric transposition) is favored for its technical simplicity, requiring only one anastomosis and providing sufficient length and perfusion, which reduces the risk of necrosis, leak, and stricture compared to other conduits, such as the colon or jejunum [9-11].

Gastric tubes, constructed as narrow, isoperistaltic, or reversed tubes, are alternatives that may be selected based on anatomical considerations. Narrow gastric tubes are associated with improved short-term functional outcomes, such as reduced reflux and thoracic stomach syndrome, compared to whole-stomach pull-ups. Still, they may have compromised vascularization at the anastomosis, increasing the risk of leaks or strictures if not carefully constructed [12,13].

Collis gastroplasty is indicated for a foreshortened esophagus to achieve a tension-free anastomosis. Modern series demonstrate that laparoscopic wedge-fundectomy Collis gastroplasty [14], often combined with fundoplication, is safe and effective, with low rates of new-onset dysphagia and good symptom control at long-term follow-up in the adult population [15].

Complications of gastric pull-up include reflux, aspiration, delayed gastric emptying, and dumping syndrome. While reflux and regurgitation are common, most patients achieve good long-term nutritional status and quality of life [16]. Total gastric transposition may have fewer leaks and strictures than partial gastric tube esophagoplasty in pediatric populations [17].

Colonic Interposition

The left transverse colonic graft is the most commonly used conduit for esophageal replacement, though the ascending or descending colon may also be used. The retrosternal esophagocolonic anastomosis was previously the most common approach, but the posterior mediastinal route has recently shown better outcomes [18].

The colonic graft should be placed in an isoperistaltic direction, though antiperistaltic placement may be used if the vascular pedicle is too short for the isoperistaltic approach. The colon is then pulled into the neck, where an end-to-side or end-to-end esophagocolic anastomosis is performed. A cologastric anastomosis is made at the cardia, with options for anti-reflux procedures such as the "Thal type" wrap or a compression method where the colon is curved against the stomach wall. Pyloroplasty may be added to facilitate gastric emptying and prevent reflux [18].

These procedures are traditionally performed via laparotomy or thoraco-laparotomy, though laparoscopically assisted techniques in children have shown promising results. A recognized complication is conduit redundancy, which can lead to stasis and dysphagia, often caused by negative pressure in the thoracic cavity. This can be minimized by avoiding pleural breaches and resecting excess colon at the cervical end. Success rates are improved when the colonic conduit is placed in the posterior mediastinum, providing a shorter and straighter path [18].

Jejunal Interposition

The jejunum can be used as a pedicled or free graft for esophageal replacement, with the staged pedicled jejunum interposition being the preferred approach. Preparing the jejunum early by ligating its vessel base promotes vascular hypertrophy and improves venous drainage. In the final stage, a pedicled graft is created by dividing mesenteric vessels near their base and isolating a jejunal segment, which is then passed through the transverse mesentery and positioned posterior to the stomach. The jejunum is anastomosed to the stomach and then to the proximal esophagus, with bowel continuity restored at the donor site [18].

A free jejunal graft, requiring microsurgical anastomosis to chest vessels, has a high failure rate, morbidity, and mortality, with reported rates of 10% mortality, 36% anastomotic leak, and up to 11% graft loss [18]. However, the jejunal conduit offers benefits such as size compatibility with the esophagus and retained peristalsis, which prevents acid reflux and avoids mediastinal compression. The primary risk is its tenuous blood supply, with complete graft necrosis potentially leading to perforation, mediastinitis, and severe complications, including death if not recognized [18].

Ileocolonic Interposition

In this procedure, the right colon is mobilized, and an appendectomy is performed. The marginal artery and vein supplying the distal terminal ileum and right colon are isolated while preserving key mesenteric vessels. Intestinal continuity is restored with an ileo-colonic anastomosis [19].

The ileocolic pedicle is then passed behind the lesser sac and stomach through the gastrohepatic vascular ligament, and a gastrocolic anastomosis is made near the greater curvature. The diaphragmatic attachments to the xiphoid are divided, and a retrosternal tunnel is created using blunt finger dissection. A cervical incision is created, and the esophagus is mobilized for adequate length [19].

The retrosternal space is entered below the sternocleidomastoid muscle, and the tunnel is enlarged to prevent compression of the ileocolic pedicle. The esophageal anastomosis is created. Drainage of the neck and anterior mediastinum is typically performed [19].

## Results

A total of 10 pediatric patients underwent foregut replacement surgery, ranging in age from seven months to 23 years. Cases included six females (n = 6, 60%) and four males (n = 4, 40%). Most patients were Hispanic (n = 8, 80%), with the remainder being non-Hispanic (n = 2, 20%). Ethnically, patients were predominantly White (n = 7, 70%), followed by Black (n = 2, 20%) and Native American/Alaska Native (n = 1, 10%). The most common indications for surgery were caustic injury (n = 3, 30%) and EA (n = 3, 30%).

A unique case involved an eight-month-old infant with severe necrotizing enterocolitis requiring multiple bowel resections. Despite multiple abdominal operations, the patient remained dependent on TPN (total parenteral nutrition) by eight months of age, prompting further evaluation. Contrast imaging demonstrated an esophagotransverse colonic fistula with only a minimal gastric remnant remaining. Given the patient's complex anatomy, definitive reconstruction was performed using a right colon interposition, creating an esophagus-colon-duodenum continuity to restore the gastrointestinal tract. In another case, a 23-year-old had previously undergone a Heller myotomy and fundoplication at the age of 15 years for achalasia. She progressively developed intractable dysphagia over the following years, eventually necessitating esophagectomy and reconstruction.

The predominant conduit type was ileocolonic interposition (n = 6, 60%), followed by gastric conduit reconstruction (n = 3, 30%) and right colon interposition (n = 1, 10%). No jejunal interpositions were performed.

Early postoperative complications included anastomotic leak (n = 5, 50%), infection (n = 3, 30%), and pneumothorax (n = 1, 10%). Late complications were more frequent, with dysphagia occurring in seven patients (n = 7, 70%), followed by stricture formation (n = 5, 50%) and GERD (n = 4, 40%). Seven patients (n = 7, 70%) had associated congenital anomalies, including atrial septal defect (ASD), ventricular septal defect (VSD), and patent ductus arteriosus (PDA) (Table 1).

**Table 1:** Postoperative outcomes of patients with esophageal replacement surgery * May require additional esophageal dilations due to the recency of the operation. ALTE: Apparent Life-Threatening Event; ASD: Atrial Septal Defect; BPD: Bronchopulmonary Dysplasia; IDA: Iron Deficiency Anemia; MDD: Major Depressive Disorder; NEC: Necrotizing Enterocolitis; PDA: Patent Ductus Arteriosus; TEF: Tracheoesophageal Fistula; TENS: Toxic Epidermal Necrolysis Syndrome; VSD: Ventricular Septal Defect

Diagnosis	Age	Conduit Type	Early Complications				Late Complications					Functional Outcomes		Congenital Anomalies
			Leak	Bleed	Infection	Other	Stricture	GERD	Dysphagia	Weight Loss	Other	Tolerating PO	No. of Dilations	
TENS, esophageal stricture	13 yrs	Ileo-colonic	No	No	No	MDD, dumping syndrome	No	Yes	Yes	Yes	Chronic diarrhea & bloating; IDA	Yes	0	None
Esophageal atresia - Type A	18 mos	Ileo-colonic	Yes	No	No	No	Yes	No	Yes	Yes	ALTE, acquired TEF	Yes	2	ASD, VSD
Esophageal atresia, stricture from caustic injury	11 yrs	Ileo-colonic, gastric	Yes	No	No	No	Yes	No	Yes	Yes	Micro-perforation & mediastinal abscess	Yes	60	VSD
Caustic injury	2 yrs	Ileo-colonic	No	No	No	No	Yes	No	Yes	No	No	Yes	16	None
Esophageal atresia - Type A	14 mos	Ileo-colonic	Yes	No	Yes (neck abscess)	No	Yes	Yes	Yes	Yes	No	Yes	4	ASD, PDA
Caustic injury	21 mos	Ileo-colonic	Yes	No	No	No	Yes	No	Yes	Yes	No	Yes	3	None
NEC, absence of gastric body	8 mos	Right colon	No	No	No	No	No	Yes	No	Yes	No	Yes	0	ASD, PDA, BPD
Progressive dysphagia (achalasia hx)	23 yrs	Ileo-colonic	No	No	Yes (SSI)	Pneumoperitoneum	No	No	No	No	No	Yes	0	None
Esophageal atresia – Type B	3 mos	Gastric	Yes	No	Yes (intrathoracic abscess)	Pneumothorax	Yes	Yes	Yes	No	No	Yes	2*	ASD, VSD, PDA
Congenital esophageal stenosis	7 yrs	Gastric	No	No	No	No	Yes	No	Yes	Yes	Intractable emesis	Yes	2	ASD

## Discussion

As previously mentioned, various conditions may necessitate esophageal replacement in the pediatric population, including long-gap EA, non-dilatable long esophageal corrosive strictures, congenital esophageal stenosis greater than 3-5 cm, and esophageal tumors. According to the literature, EA is the most common indication for replacement, followed by corrosive injury, which matches our experience [20]. In addition, some of our patients presented with congenital anomalies such as ASD, VSD, and PDA. The American Heart Association emphasizes that congenital heart disease in children undergoing noncardiac surgery, including esophageal replacement, raises the risk of perioperative morbidity and mortality, necessitating comprehensive preoperative cardiac evaluation, optimization of hemodynamics, and tailored anesthetic planning. These patients may have altered physiology, increased susceptibility to fluid shifts, and a higher risk of complications such as arrhythmia, hypoxemia, and hemodynamic instability [21].

Previously, options for esophageal replacement were discussed. Each technique has advantages and disadvantages, and the choice depends on patient anatomy, available structures, and surgical expertise. In our series, the ileocolic approach was preferred in 60% of cases due to its technical advantages and surgeon experience with the procedure. Our experience with colonic interposition and gastric interposition was limited, with both patients developing gastroesophageal reflux. No jejunal interposition was performed. The decision to avoid jejunal grafts was based on the complexity of microvascular anastomosis, increased operative time, and concern for graft ischemia in our pediatric population. Overall, conduit selection remains individualized, but our outcomes support the ileocolic graft as a reliable and well-tolerated option in this cohort.

Complications were categorized as early and late. We had no intraoperative complications. The most common early postoperative complications included anastomotic leak and wound infection. The incidence of anastomotic leak in our series was higher than that reported in the literature. This could be attributed to the complexity of the underlying pathology, the use of ileo-colonic conduits, and the small sample size of our cohort. Additionally, patient factors such as nutritional status and comorbidities may have contributed to impaired healing. Late complications primarily included dysphagia, stricture formation, and GERD, consistent with previous reports [18-20,22-24]. Careful patient selection, perioperative optimization, and standardized postoperative management may help mitigate these risks in future cases.

Mortality rates following esophageal replacement in the pediatric population are generally reported in the range of 2-13%, with variation depending on disease severity, surgical expertise, and available intensive care resources. Large multicenter and single-center studies indicate that early postoperative mortality is typically low in high-resource, experienced centers, with 30-day mortality rates around 2.8-3%. However, higher mortality rates have been observed in settings with limited resources or in series including more complex or delayed presentations [22-24]. In our series, no mortality was reported. These findings highlight the importance of meticulous surgical technique, close postoperative monitoring, and long-term follow-up to optimize outcomes in pediatric esophageal replacement surgery.

This study is limited by its retrospective design and single-center and single-surgeon experience, which may limit generalizability. Cases were also performed in an academic center in a rural setting. The proximity of our center to a major U.S. city with larger freestanding pediatric hospitals likely affected our catchment over the 11-year period and is reflected in the small sample size. There are no deaths in our series; however, this may again be attributed to the small sample. Additionally, the heterogeneity in patient indications, conduit types, and surgical techniques over the 11-year period introduces potential variability in outcomes.

## Conclusions

Although uncommon, foregut replacement remains an important reconstructive option for select pediatric patients with complex esophageal pathology. Careful preoperative assessment, individualized conduit selection, and meticulous surgical planning are essential to optimize functional outcomes and minimize postoperative complications. Long-term follow-up remains critical given the frequency of late complications such as dysphagia, stricture formation, and gastroesophageal reflux.
